# Curcumin regulates gene expression of insulin like growth factor, B-cell CLL/lymphoma 2 and antioxidant enzymes in streptozotocin induced diabetic rats

**DOI:** 10.1186/1472-6882-13-368

**Published:** 2013-12-24

**Authors:** Sabry M El-Bahr

**Affiliations:** 1 Department of Physiology, Biochemistry and Pharmacology (Biochemistry), College of Veterinary Medicine and Animal Resources, King Faisal University, Al Hofuf, Al-Ahsa, Saudi Arabia; 2 Department of Biochemistry, Faculty of Veterinary Medicine, Alexandria University, Alexandria, Egypt

**Keywords:** Diabetes mellitus, Antioxidant enzymes, Bcl-2, Insulin like growth factor, PCR, Gene expression

## Abstract

**Background:**

The effects of curcumin on the activities and gene expression of antioxidant enzymes, superoxide dismutase (SOD), catalase (CAT), glutathione peroxidase (GPX), glutathione-S-transferase (G-ST), B-cell CLL/lymphoma 2 (Bcl-2) and insulin like growth factor-1 (IGF-1) in diabetic rats were studied.

**Methods:**

Twenty four rats were assigned to three groups (8 rats for each). Rats of first group were non diabetic and rats of the second group were rendered diabetic by streptozotocin (STZ). Both groups received vehicle, corn oil only (5 ml/kg body weight) and served as negative and positive controls, respectively. Rats of the third group were rendered diabetic and received oral curcumin dissolved in corn oil at a dose of 15 mg/5 ml/kg body weight for 6 weeks.

**Results:**

Diabetic rats showed significant increase of blood glucose, thiobarbituric acid reactive substances (TBARS) and activities of all antioxidant enzymes with significant reduction of reduced glutathione (GSH) compare to the control non diabetic group. Gene expression of Bcl2, SOD, CAT, GPX and GST was increased significantly in diabetic untreated rats compare to the control non diabetic group. The administration of curcumin to diabetic rats normalized significantly their blood sugar level and TBARS values and increased the activities of all antioxidant enzymes and GSH concentration. In addition, curcumin treated rats showed significant increase in gene expression of IGF-1, Bcl2, SOD and GST compare to non diabetic and diabetic untreated rats.

**Conclusion:**

Curcumin was antidiabetic therapy, induced hypoglycemia by up-regulation of IGF-1 gene and ameliorate the diabetes induced oxidative stress via increasing the availability of GSH, increasing the activities and gene expression of antioxidant enzymes and Bcl2. Further studies are required to investigate the actual mechanism of action of curcumin regarding the up regulation of gene expression of examined parameters.

## Background

Diabetes is one of the most common chronic diseases affecting more than 100 million people world wide. The two major types of diabetes mellitus are characterized by hyperglycemia, abnormal lipid and protein metabolism along with specific long term complications affecting the retina, kidney and nervous system [1]. Hyperglycemia is an important factor in the development and progression of the complications of diabetes mellitus [2]. Hyperglycemia caused an increase in glucose autoxidation, protein glycation and the subsequent oxidative degradation of glycated protein leads to enhanced production of reactive oxygen species (ROS) [3]. The levels of ROS are regulated by a variety of cellular defense mechanisms consisting of enzymic and non-enzymic antioxidants [4]. The primary scavenger enzymes involved in detoxifying ROS in mammalian systems are CAT, SOD, GPX and GST [5]. The reports in the literature regarding the effect of diabetes-induced hyperglycemia on antioxidant enzymes are contradictory. They have been reported to decrease, increase or remain unaltered in diabetic animals with wide variations depending on age of the animal or duration of diabetes [6-8] or tissues examined [9,10]. These discrepancies may arise due to variations in enzyme activity over time (e.g. compensatory increases in enzyme activity to overcome raised oxidative stress or direct inhibitory effects of ROS as well as due to the type of tissue under examination). High levels and/or inadequate removal of ROS may cause severe metabolic imbalance and oxidative damage to biological macromolecules named oxidative stress [5]. Oxidative stress often causes cell death via apoptosis that is regulated by certain functional genes and their protein products. Bcl-2, which is an integral mitochondrial membrane protein, blocks apoptosis induced by a wide array of death signals and is involved in decreasing ROS production [11]. Bcl-2 protein itself has antioxidant ability either by prevention of entrance of cytochrome *c* to the cytosol or by binding directly with cytochrome c to reduce generation of free radicals [12]. Furthermore, Bcl-2-overexpressing cells exhibit elevated expression of antioxidant enzymes and higher levels of cellular GSH [13]. IGF-1 is a peptide hormone and has structural homology with insulin. Liver is the main source of circulating IGF-1 [14]. IGF-1 is widely present in tissues of mammalian animals and has a number of bioactivities including regulation of metabolism and enhancement of growth and development of tissues [15]. IGF-1 may probably be involved in the metabolic abnormalities and complications associated with diabetes [16]. IGF-1 maintain euglycemia by increasing the peripheral uptake of glucose and fatty acids and decreasing the insulin resistance [17]. IGF-1 augments the peripheral glucose uptake via binding to normal IGF-1 receptors mainly in muscle tissues. However, with worsening of insulin resistance, the expression of hybrid receptors in muscle and adipose tissues are increased and leads to increased glucose and fatty acids uptake in these tissues [17]. The effect of IGF-1 on pancreatic β-cells also was also documented. β-cells express the IGF-1 receptors and tyrosine kinase activity of these receptors on the insulin receptor substrate pathway could potentially alter insulin secretion by influencing cell replication and survival [17].

There are many anti-diabetic plants, which might provide useful sources for the development of drugs, in the treatment of diabetes mellitus. The literature on medicinal plants with hypoglycemic activity is vast. As many of these plants were used for many centuries and some times as regular constituents of the diet, it is assumed that they do not have many side effects. However chronic consumption of large amounts of traditional remedies must always be taken with caution as toxicity studies have not been conducted for most of these plants [18,19]. Curcumin, the active ingredient from the spice turmeric is a potent antioxidant and anti-inflammatory agent with hepatoprotective, anticarcinogenic and antimicrobial properties [20,21]. Curcumin also has a beneficial effect on blood glucose in diabetics and increases gastric mucosal secretion in rabbits [22]. The Turmeric rhizomes have been reported to possess antidiabetic properties as its alcohol extract possesses active constituents showing blood glucose lowering activity in alloxan induced diabetic rats [18]. Although, the antidiabetic effects of curcumin have been extensively studied on peripheral blood [23], the literature reports are still contradictory. In addition, the molecular studies regarding the effect of curcumin on gene expression of antioxidant enzymes have not been clearly elucidated. Additionally, the effect of curcumin on gene expression of Bcl-2 and IGF-1 was not determined so far. Upon this basis, streptozotocin diabetic rats were used in the present study to evaluate the effects of curcumin on activities and gene expression of antioxidant enzymes. Furthermore, the effect of curcumin on gene expression of Bcl-2 and IGF-1 in streptozotocin-induced diabetes in rats was also determined.

## Methods

### Chemicals

Curcumin, streptozotocin, agarose, ethidium bromide, chloroform, and isopropanol were purchased from Sigma Chemical Co., MO, USA. The glucotest strips were supplied by Roche, USA. The DNA ladder was purchased from MBI, Fermentas, USA. Primers were purchased from metabion international Ag, Germany. One step RT-PCR kits were purchased from Qiagen Germany. TriZol reagent was supplied by Invitrogen, Carlsbad, CA. QuantiFast™ SYBR Green PCR Master Mix kit was supplied by QIAGEN, Hilden; Germany. All other chemicals were of the highest analytical grade.

### Experimental animals

A total of twenty four adult albino rats weighing between 200 ± 15 g were maintained as performed by national guidelines and protocols, approved by the King Faisal University Scientific Research Ethics Committee. They were housed in clean and disinfected cages. Commercial basal diet and water were provided *ad libitum*. Rats were subjected to natural photoperiod of 12 hr light:dark cycle throughout the experimental period (6 weeks). All rats received basal diet for two weeks before the start of the experiment for adaptation and to ensure normal growth and behavior.

### Induction of experimental diabetes

Diabetes was induced by administering intraperitonial injection of a freshly prepared solution of STZ (60 mg/kg b. w.) in 0.1 M cold citrate buffer (pH 4.5) to the overnight fasted rats [24]. Because of the instability of STZ in aqueous media, the solution was made using cold citrate buffer (pH 4.5) immediately before administration. Control rats were injected with citrate buffer alone. The rats were allowed to drink 5% glucose solution overnight to overcome the drug-induced hypoglycemia. After 72 hours, fasting blood glucose levels were monitored. Animals having blood glucose levels 145 mg/dl were excluded from the experiment and animals having blood glucose values above 250 mg/dl on the third day after STZ injection were considered as diabetic rats [25]. Then the treatment was started on the third day after STZ injection and it was considered as first day of treatment.

### Experimental design

Rats were divided into three groups (8 rats for each). The first group (group I) served as control and received vehicle, corn oil only (5 ml/kg body weight). Rats of the second group (group II) rendered diabetic by administering intraperitonial injection of a freshly prepared solution of STZ (60 mg/kg b. w.) in 0.1 M cold citrate buffer (pH 4.5) to the overnight fasted rats [24]. Rats of the third group (group III) rendered diabetic and received curcumin dissolved in corn oil at a dose of 15 mg/5 ml/kg body weight for 6 weeks by gastric gavages [26,27].

### Samples collection and blood glucose level estimation

Blood samples were collected from the fasted rats of three groups prior to the treatment with curcumin and four times after oral administration of the treatments up to 6 weeks (first, second, fourth and sixth weeks). Blood samples were collected by snipping tail with sharp razor and blood glucose level was then measured immediately by glucose strips (haemo-glucotest). At the end of the experiment, liver samples were collected from all groups, washed by normal saline solution, dried by towel, flash frozen in liquid nitrogen and subsequently frozen at –80°C until the time of analysis of oxidative stress biomarkers, RT-PCR and real time RT-PCR.

### Determination of hepatic antioxidant enzymes, thiobarbituric acid reactive substances and reduced glutathione

One gram of liver tissues was homogenized in 5 ml of cold 20 mM HEPES buffer, pH 7.2, containing 1 mM EGTA, 210 mM mannitol and 70 mM sucrose. After centrifugation (1500 × g/5 minute at 4°C, the supernatant was removed and stored frozen at –80°C until the time of analysis of SOD. Another one gram of liver tissues was homogenized in 5 ml of cold buffer of 50 mM potassium phosphate buffer, pH 7, containing 1 mM EDTA. After centrifugation at 10.000 × g/15 minutes at 4°C, the supernatant was removed and stored frozen at –80°C until the time of analysis of CAT, GPX, GST, GR and GSH. The extent of lipid peroxidation in terms of thiobarbituric acid reactive substances (TBARS) formation was measured by mixing one gram of liver tissues with RIPA buffer (Item No. 10010263, Cayman chemical company, USA). After homogenization, sonication and centrifugation (1600 × g/10 minutes), the supernatant was removed and stored frozen at -80°C until the time of analysis. The activities of CAT (nmol/min/gram tissue; Cayman Chemical Company, USA, Catalog No. 707002), GPX (nmol/min/ gram tissue; Cayman Chemical Company, USA, Catalog No. 703102), SOD (U/gram tissue; Cayman Chemical Company, USA, Catalog No. 706002), GST (nmol/min/ gram tissue; Cayman Chemical Company, USA, Catalog No. 703302) and concentrations of GSH (μM; Cayman Chemical Company, USA, Catalog No. 703002) and TBARS (μM; Cayman Chemical Company, USA, Catalog No. 10009055) were determined by ELISA reader (Absorbance Microplate Reader ELx 800TM BioTek®, USA). Results were calculated according to the manufacture instructions.

### Total RNA isolation and RT-PCR analysis of hepatic antioxidant enzymes, Bcl-2 and IGF-1

The Primers of GPX, CAT and SOD [28] as well as IGF-1 and β-actin genes [29] and Bcl-2 [30] were taken from literature. The primers were checked for their Tm values, hairpin loops, dimers, cross- dimers and number of repeats and runs using Net Primer (Oligoanalyzer 3.1). β-actin gene was used as an internal standard (house keeping gene). Frozen liver samples (approximately 1 g per sample) were immediately added to lysis buffer (Qiagen, Germany) and homogenized using homogenizer (Tissue Ruptor, Qiagen GmbH, Germany). Reverse transcriptase polymerase chain reaction (RT-PCR) was performed with Qiagen one-step RT-PCR kit (Qiagen, Germany) according to the manufacturer’s instructions. The purity of RNA at 260/280 OD ratio and RNA integrity was evaluated using Multi-Mode Microplate reader (SYNERGY Mx, BIO-TEK. Winooski, Vermont, USA). Only high purity samples (OD260/280 >1.8) were subjected to further manipulation. The master mix was prepared according to the manufacture instruction. The whole volume of the reaction was 25 μl for each gene of interest arranged as 5 μl of 5× buffer, 5 μl of Q-buffer, 1 μl of dTNPs, 1 μl of forward primer, 1 μl of the reverse primer, 1 μl of enzyme mix (reverse transcriptase and Taq polymerase), 6 μl of nuclease free water and finally 5 μl of RNA. The reaction has been done in a Bio-Rad thermal cycler (MyCycler, Germany). The RT-PCR conditions were as follows: (1) reverse transcription, 30 min, 50°C, (2) initial PCR activation step, 15 min, 95°C, (3) 3-step cycling for 40 cycles, each cycle consisting of denaturation for 30 sec at 94°C followed by annealing for 30 sec at 52-57°C (according to gene of interest as described in Table 1) and extension for 1 min at 72°C. The template concentration and the cycle number were optimized to ensure linearity of response and to avoid saturation of the reaction (40 cycle was better). The PCR products were then resolved on 1.5% agarose gels. The bands were identified based on the product size using a 5000 bp DNA ladder; Documented using a Gel documentation system (Gel Doc^TM^XR System, Bio-Rad) and the prints were scanned. The scanned images were quantified densitometrically with the aid of NIH image program (http://rsb.info.nih.gov/nih-image/). The results were normalized to the levels obtained for the β-actin gene by taking a ratio of the value obtained for the gene of interest to that of β-actin and then relative to the control.

**Table 1:** Details giving primer sequences and expected product size for the genes amplified

**cDNA**	**Sequence**	**Annealing temp.**	**Number of cycles**	**RT**-**PCR product size**
**β**-**actin F**	5^/^-AGC CAT GTA CGT AGC CAT CC-3^/^	55	40	230
**β**-**actin R**	5^/^- CTC TCA GCT GTG GTG GTG AA-3^/^			
**IGF1F**	5^/^- CTG GGT GTC CAA ATG TAA CT-3^/^	52	40	170
**IGF1R**	5^/^-GTA TCT TTA TTG GAG GTG CG-3^/^			
**SODF**	5^/^- AGG ATT AAC TGA AGG CGA GCA T-3^/^	55	40	410
**SODR**	5^/^- TCT ACA GTT AGC AGG CCA GCA G-3^/^			
**CATF**	5^/^-ACG AGA TGG CAC ACT TTG ACA G −3^/^	55	40	341
**CATR**	5^/^-TGG GTT TCT CTT CTG GCT ATG G-3^/^			
**GPxF**	5^/^-AAG GTG CTG CTC ATT GAG AAT G-3^/^	57	40	406
**GPxR**	5^/^-CGT CTG GAC CTA CCA GGA ACT T-3^/^			
**Bcl**-**2 F**	5^/^- TCC ATT ATA AGC TGT CAC AGA GG-3^/^	55	40	350
**Bcl**-**2R**	5^/^- GAA GAG TTC CTC CAC CAC C-3^/^			
**GSTF**	5^/^- GCT GGA GTG GAG TTT GAA GAA-3^/^	55	40	575
**GSTR**	5/- GTC CTG ACC ACG TCA ACA TAG-3^/^			

### Total RNA isolation and Real time RT-PCR analysis of hepatic antioxidant enzymes, Bcl-2 and IGF-1

Liver tissues (approximately 1 g of tissue per sample) were immediately added to 1 ml of TriZol reagent (Invitrogen, Carlsbad, CA) and homogenized using homogenizer (Tissue Ruptor, Qiagen GmbH, Germany). One milliliter of the tissue homogenate was transferred to a microfuge tube and total RNA was extracted by adding 0.2 ml chloroform. Afterwards, samples were vortexed vigorously for 15 seconds and incubated at room temperatuer for 3 minutes. After centrifugation (12,000 g/15 minutes) at 4°C, the aqueous phase containing RNA was transfered into new tubes. RNA was precipited by mixing the aqueous phase with 0.5 ml isopropyl alcohol and incubated at room temperuter for 10 minutes. After centrifugation at 12,000 g for 10 minutes at 4°C, RNA pellets were washed by mixing and vortexing with 1 ml of 75% ethanol. After centrifugation (7.500 g/5 minutes) at 4°C, RNA pellets were resuspended in nuclease free water (Life Technologies. USA). The purity of RNA at 260/280 OD ratio and RNA integrity has been estimated as described above in RT-PCR analysis. cDNA was prepared from RNA samples according to Revers Transcription System Kit (Promega, Madison, USA) by using Bio-Rad Thermal Cycler (T100™, Foster city, California, USA). Briefly, total RNA were activated at 70°C for 10 minutes and 20 μl reaction mix were made of 4 μl MgCl_2_, 2 μl of reverse transcription 10× buffer, 2 μl of dNTP mixture (10 mM), 0.5 μl of random primers, 0.75 μl of AMV reverse transcriptase enzyme, 1 ng RNA and nuclease-free water to a final volume of 20 μl. Then the reaction was incubated at 42°C for 60 minutes followed by incubation at 94°C for 5 minutes. cDNA was diluted up to 100 μl with nuclease-free water for PCR amplification. Real time RT-PCR was performed using QuantiFast™ SYBR Green PCR Master Mix kit (QIAGEN, Hilden; Germany). The 25 μl reaction for each examined gene was prepared from 12.5 μl of master mix; 2 μl forwerd primer (10 pmol); 2 μl revers primer (10 pmol); 2 μl cDNA of the sample and 6.5 μl of nuclease-free water. Cycling parameters were, 50°C for 2 minutes, 95°C for 15 minutes, 40 cycles of 95°C for 10 seconds, followed by 55°C for 30 seconds and 72°C for 10 seconds with final melting at 95°C for 20 seconds. For each gene examined, duplicate samples from each cDNA analyzed by real time RT-PCR using the Bio-Rad CFX Manager 3.0 Software of the C1000 Touch thermal cycler-CFX96 Real time PCR (BIO-RAD, Foster city, California, USA). The β-actin mRNA fragment was used as housekeeping gene to normalize the expression data. The primer sequences are the same of conventional PCR technique as described above (Table 1).

### Statistical analysis

All data was presented as mean ± standard error of mean by using student-t test. All tests were performed using computer package of the statistical analysis system [31]. The relative gene expression of target genes in comparison to the β-actin reference gene was calculated using the Bio-Rad CFX Manager 3.0 Software of the C1000 Touch thermal cycler-CFX96 Real time PCR (BIO-RAD, Foster city, California, USA).

## Results

### Blood glucose level estimation

The results for the effects of curcumin on blood glucose concentration of STZ diabetic rats are shown in Table 2. At the start of the experiment or at zero time, there were no statistically significant (*P* > 0.05) differences in the mean values of blood glucose level between all experimental groups. After injection of STZ, the mean values of blood glucose levels in untreated diabetic rats (group II) were remained above 330 mg/dl during the entire period of the study, which were significantly (*P* < 0.05) higher than those of the non diabetic normal control rats by approximately 230%. The percentage of increase in glucose level of diabetic rats (230%) was reduced by 39.7% (199 mg/dl) as result of curcumin administration.

**Table 2:** **Effect of oral administration of curcumin at the start and during six weeks of the experiment on blood glucose concentration** (**mg**/**dl**) **in streptozotocin diabetic rats**

**Groups**	**Blood glucose concentration (mg/dl)**				
	**At the start**	**1**^**st **^**week**	**2**^**nd **^**week**	**4**^**th **^**week**	**6**^**th **^**week**
I	100 ± 1.1	100 ± 2.1	96 ± 1.2	92 ± 1.1	90 ± 2.1
II	102 ± 1.2	366 ± 1.9*	330 ± 1.5*	340 ± 1.4*	350 ± 1.7*
III	98.2 ± 2.1	199 ± 1.1**	187 ± 1.0**	192 ± 1.3**	197 ± 1.2**

I (non diabetic), II (diabetic), III (diabetic treated with curcumin); Values are mean ± SD of 8 rats; *Mean values are significantly (P < 0.05) different compare to the control (group I). **Mean values are significantly (P < 0.05) different compare to diabetic rats (group II).

### Hepatic antioxidant enzymes, thiobarbituric acid reactive substances and reduced glutathione

The results for the effect of curcumin on lipid peroxidation, GSH and antioxidant enzyme activities of STZ diabetic rats are illustrated in Table 3. The current results revealed that, lipid peroxidation (TBARS values) increased significantly (*P* < 0.05) by 19.3% in diabetic untreated rats (42.9 ± 0.1 μM) compare to control non diabetic rats (34.6 ± 0.05 μM). However, administration of curcumin to diabetic rats normalized these TBARS values (32.6 ± 0.09 μM). In diabetic untreated rats, the activities of CAT (32.6 ± 0.2 nmol/min/gm tissues), SOD (7.2 ± 0.1 U/gm tissue), GPX (220.4 ± 2.1 nmol/min/gm tissues) and GST (175.2 ± 2.0 nmol/min/gm tissue) enzymes were increased significantly (*P* < 0.05) compare to control (23.2 ± 0.1 nmol/min/gm tissues; 6.1 ± 0.03 U/gm tissue; 209 ± 1.2 nmol/min/gm tissues; 163.0 ± 1.1 nmol/min/gm tissue), respectively. With the exception of CAT, the administration of curcumin caused significant (*P* < 0.05) increase in the activities of these enzymes (SOD: 8.3 ± 0.07 U/gm tissue; GPX: 323 ± 3.2 nmol/min/gm tissues; GST: 260.1 ± 3.1 nmol/min/gm tissues) compare to diabetic untreated rats. It is statistically obvious that, the activities of SOD, GPX and GST were increased significantly (*P* < 0.05) approximately by 13.3%, 31.8% and 32.6%, respectively in diabetic rats treated with curcumin compare to control non diabetic rats and increased significantly (*P* < 0.05) approximately by 26.5%, 35.3% and 37.3%, respectively in diabetic rats treated with curcumin compare to diabetic untreated rats. The concentration of GSH was decreased significantly (*P* < 0.05) in diabetic untreated rats (3.1 ± 0.1 μM) compare to control non diabetic rats (4.8 ± 0.2 μM). Interestingly, The concentration of GSH was increased significantly by approximately 44.8% and 64.4% in diabetic rats treated with curcumin compared with nondiabetic and diabetic untreated rats, respectively.

**Table 3:** **Effect of oral administration of curcumin for six weeks on lipid peroxidation**, **reduced glutathione and antioxidant enzyme activities in streptozotocin induced diabetic rats**

	**Group I**	**Group II**	**Group III**
TBARS (μM)	34.6 ± 0.05	42.9 ± 0.1*	32.6 ± 0.09^#^
CAT (nmol/min/gram tissue)	23.2 ± 0.10	32.6 ± 0.2*	31.1 ± 1.50*
SOD (U/gram tissue)	6.1 ± 0.03	7.2 ± 0.1*	8.3 ± 0.07*^#^
GPX (nmol/min/ gram tissue)	209.0 ± 1.2	220.4 ± 2.1*	323.0 ± 3.20*^#^
GST (nmol/min/ gram tissue)	163.0 ± 1.1	175.2 ± 2.0*	260.1 ± 3.10*^#^
GSH (μM)	4.8 ± 0.2	3.1 ± 0.1*	8.7 ± 0.10*^#^

Group I (non diabetic), group II (diabetic), group III (diabetic treated with curcumin); Values are mean ± SD of 8 rats; *TBARS*: thiobarbituric acid reactive substances; *CAT*: catalase; *SOD*: superoxide dismutase; *GST*: glutathione-S transferase; *GSH*: reduced glutathione.

*Mean values are significantly (P < 0.05) different compare to the control (group I). ^#^Mean values are significantly (P < 0.05) different compare to diabetic rats (group II).

### RT-PCR analysis of hepatic antioxidant enzymes, Bcl-2 and IGF-1

**Figure 1:**
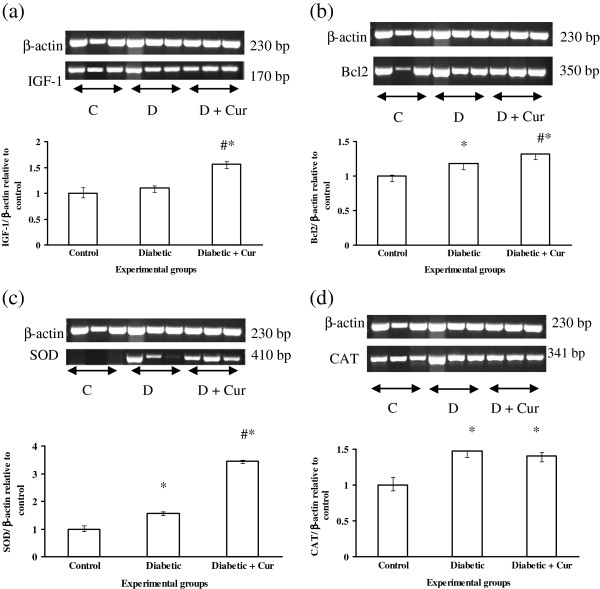
**RT-PCR analysis of IGF-1 (a), Bcl2 (b), SOD (c), CAT (d) in liver tissues of control non diabetic (C), experimentally diabetic (D) and Curcumin (Cur) treated diabetic rats.** RT-PCR was performed with Qiagen one-step. The densitometric analysis of the expresses bands (lower columns) was normalized with that of β-actin and then calculated as relative to the control group. Values are expressed as mean ± SEM for 8 rats. ^*^Values are significant different (p < 0.5) compared to control non diabetic group. ^#^Values are significant different (p < 0.5) compared to control diabetic group.

**Figure 2:**
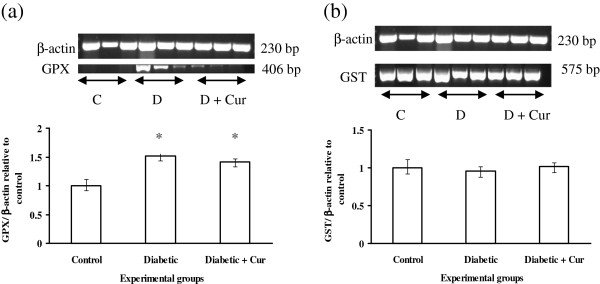
**RT**-**PCR analysis of GPX (a) and GST (b) in liver tissues of control non diabetic**, **experimentally diabetic and curcumin (Cur) treated diabetic rats.** RT-PCR was performed with Qiagen one-step. The densitometric analysis of the expresses bands (lower columns) was normalized with that of β-actin and then calculated as relative to the control group. Values are expressed as mean ± SEM for 8 rats. *Values are significant different (p < 0.5) compared to control non diabetic group. #Values are significant different (p < 0.5) compared to control diabetic group.

The RT-PCR data as showed in Figure 1a indicated that, experimental diabetes by STZ did not affect (p > 0.05) the gene expression of IGF-1 compare to control non diabetic rats. Over expression of this gene was induced when diabetic rats treated with curcumin compare to control non diabetic and diabetic untreated rats. RT-PCR results revealed also that, experimental diabetes induced significant (*P* < 0.05) increase in the gene expression of Bcl2, SOD, CAT and GPX as explained in Figure 1b, 1c, 1d and Figure 2a, respectively without significant effect on gene expression of GST (Figure 2b). The RT-PCR data with regards to effect of curcumin indicated that, curcumin administration induced significant increase in mRNA expression of Bcl2 (Figure 1b) and SOD (Figure 1c) compare to control non diabetic and diabetic untreated rats. In addition, curcumin induced up-regulation of CAT and GPX gene expression compare to control non diabetic rats but remained comparable to that induced by STZ compare to diabetic rats.

### Real time RT-PCR analysis of hepatic antioxidant enzymes, Bcl-2 and IGF-1

**Figure 3:**
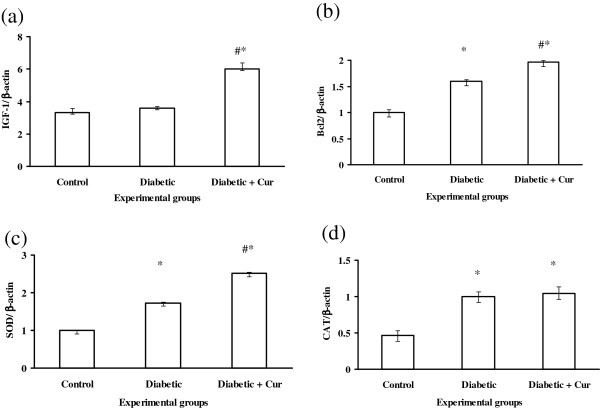
**Real time RT-PCR analysis of IGF**-**1 (a)**, **Bcl2** (**b**), **SOD (c)**, **CAT** (**d**) **in liver tissues of control non diabetic (C)**, **experimentally diabetic (D) and Curcumin** (**Cur**) **treated diabetic rats.** Values are expressed as mean ± SEM for 8 rats. ^*^Values are significant different (p < 0.5) compared to control non diabetic group. ^#^Values are significant different (p < 0.5) compared to control diabetic group.

**Figure 4:**
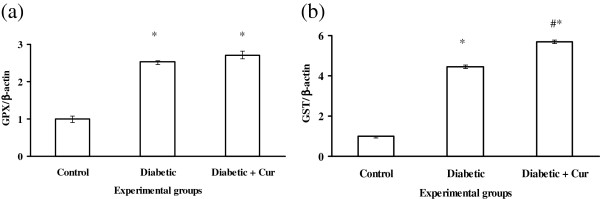
**Real time RT-PCR analysis of GPX (a) and GST (b) in liver tissues of control non diabetic**, **experimentally diabetic and curcumin** (**Cur**) **treated diabetic rats.** Values are expressed as mean ± SEM for 8 rats. *Values are significant different (p < 0.5) compared to control non diabetic control. #Values are significant different (p < 0.5) compared to control diabetic group.

The real time PCR analysis was parallel to that observed by RT-PCR for gene expression of IGF-1, Bcl2, SOD, CAT and GPX (Figures 3 and 4). However, real time analysis indicated that, experimental diabetes by STZ increased significantly (p < 0.05) the gene expression of GST compare to control non diabetic rats (Figure 4b) and curcumin administration induced significant increase in the expression of this gene (Figure 4b) compare to control non diabetic and diabetic untreated rats.

## Discussion

The present findings revealed that, injection of STZ (60 mg/kg b.wt.) induced significant hyperglycemia in rats. This dose caused damage to β-cells of the islets of langerhans and emergence of clinical diabetes within 2–4 days as results of autoimmune process [32]. Similar to the present findings, previous articles [33,34] reported that, STZ induced significant hyperglycemia. Hyperglycemia may be attributed to enhancement of gluconeogenesis as a result of absence of insulin [35]. The hypoglycemic effect of curcumin observed in the current study comes in accordance with those obtained in rats [22,36]. Authors reported that curcumin had a beneficial effect on blood glucose in diabetes and increases gastric mucosal secretion. Dietary inclusion of curcumin (0.6-0.9 g/kg diet), improved the adverse effect of aflatoxins in blood glucose values of broiler chickens [37]. Similar hypoglycemic effect of curcumin [23] and turmeric rhizomes [18] was observed in alloxan induced diabetes in rats. The medicinal plants exert its hypoglycemic effect either directly on the pancreas and subsequent release of insulin or indirectly via regulation of hepatic enzymes of glycolysis and gluconeogenesis [22]. The hypoglycemic effect of curcumin may be attributed to stimulation of polyol pathway in which glucose converted to sorbitol by the aids of aldose reductase in the presence of NADPH. This pathway beside its lowering effect of glucose, it enhances the glutathione redox cycle and subsequent protection against ROS [38]. This was underlined by the results obtained in the present study indicating significant increase of GSH contents in diabetic rats treated with curcumin.

The significant increase in lipid peroxidation in diabetic rats by 19.3% indicated significant oxidative stress. The present data of lipid peroxidation come in accordance with the findings of Kakkar et al. [3] and Limaye et al. [39] who reported a 60 and 78.45% increase at six weeks of streptozotocin induced diabetes, respectively. The present findings are in accordance also with previous report [40] indicated significant increase in TBARS in STZ diabetic rats. The lipid peroxidation may attribute to the hypoinsulinemia caused by STZ progressive deterioration of normal pancreatic β-cell function. This hypoinsulinemia induced an increase in the activity of fatty acyl Co-A oxidase that initiate the β-oxidation of fatty acids resulting in lipid peroxidation [41,42]. The present study showed that, administration of curcumin significantly normalized the liver TBARS in diabetic rats. These results are underlined by the significant increase in concentrations of GSH and activities and expression of antioxidant enzymes in curcumin treated diabetic rats. The whole mechanism can be discussed briefly as fellow: STZ caused damage to β-cells of pancreas and led to production of ROS and absence of insulin. Hyperglycemia and lipid peroxidation were produced as a result of absence of insulin and high level of ROS, respectively [35]. Superoxide radicals, hydrogen peroxide and hydroxyl radicals are the main ROS which caused damage to DNA, proteins and other macromolecules and subsequent diabetic complications. The natural antioxidant system consists of numerous antioxidant compounds and several antioxidant enzymes such as SOD, CAT, GPX and GST. The primary ROS produced in the aerobic organisms is superoxide radical that is a highly reactive cytotoxic agent. Superoxide radical is converted to H_2_O_2_ by SOD. Further, H_2_O_2_ in turn, is converted to molecular oxygen and H_2_O by either CAT or GPX. Moreover, GPX can reduce lipid peroxides and other organic hydroperoxides that are highly cytotoxic products. Thus, SOD, CAT and GPX constitute the principal components of the antioxidant system and their deficiencies can cause oxidative stress. In addition, GSH conjugated with xenobiotic substances by the aids of GST. Literature with regards to the effect of oxidative stress on antioxidant enzyme activities varied significantly from no changes [7], decrease [22,36,43] and increase [39,44,45] depending on the duration of the experiments and the age of animals. When ROS concentrations overwhelms the antioxidant capacity, oxidative stress is generated with characteristic cell damage. In the present study, GSH concentration was decreased significantly in STZ diabetic rats because it was consumed for conjugation with STZ, a process catalyzed by GST [22,36]. A significant increase of GST activity has been observed in STZ diabetic rats to facilitate the conjugation process between STZ and GSH [22,36]. In addition, activities of all antioxidant enzymes were also increased significantly in these diabetic rats to scavenge the ROS and avoid diabetic complications. Administration of curcumin to diabetic rats increased GSH and augmented the increase of antioxidant enzymes activities and even gene expression of these enzymes to counteract the oxidative stress. the mechanism applied for enhancement of gene expression of antioxidant enzymes is beyond the objectives of the current study [22,36,40,43,46]. The present findings indicated that, the activities of antioxidant enzymes were not increased in normal non diabetic rats because these rats were normal, not diabetic and hence ROS were not generated and no needs for activation of antioxidant system. The situation was differed in positive control diabetic rats as STZ destroyed the beta cell of pancreas, no insulin, high ROS and increasing the activity and even gene expression of antioxidant enzymes is essential to counteract the stressful situations. CAT was increased significantly in diabetic untreated rats to counteract the increased amount of H_2_O_2_. Like CAT, the GPX and SOD were also increased significantly in diabetic rats to reduce the lipid peroxides. Similar results [7,8,39] reported an increase in the activities of GPX of diabetic rats. Parallel to the present study, the increased activities of SOD in the liver, heart and pancreas was observed in STZ diabetic rats [7] and diabetic patients [9]. In the contrary, other works [22,36,43] reported a decrease in the above mentioned antioxidant enzyme activities in diabetic rats, however the mRNA levels have not been examined. Interestingly, curcumin induced significant elevation of all examined antioxidant enzymes activities in diabetic rats compare to non treated diabetic rats and non diabetic control rats of the present experiment. This may attributed to increased availability of GSH in diabetic rats treated with curcumin compare to non treated diabetic rats and diabetic rats. Similar results were obtained in STZ induced diabetes in rats and treated with curcumin [22,36,43].

Previous report [39] concluded that, STZ elevated the activities of antioxidant enzymes by increasing their gene expression. However, the authors depend on semi-quantitative PCR analysis. The present study showed that, there are positive correlation between gene expression and activities of the antioxidant enzymes in STZ diabetic rats. Moreover, the gene expression was evaluated by both conventional and relative quantitative real time RT-PCR. The real time RT-PCR was performed for confirmation of the conventional RT-PCR results. In addition, the real time RT-PCR was useful and sensitive than conventional RT-PCR for GST gene expression analysis. Combining the results of conventional and real time RT-PCR, STZ induced elevation of mRNA expression of all antioxidant enzymes and Bcl2. Interestingly, curcumin increased significantly the gene expression of theses parameters with significant increase also in gene expression of IGF-1 in STZ diabetic rats compare to untreated diabetic rats and non diabetic rats. The significant increase of SOD, CAT, GPX and GST gene expression in STZ diabetic rats seems to be a natural response of the cell to cope with oxidative stress. The significant increase of gene expression of these antioxidant enzymes in diabetic rats treated with curcumin acts as a supplier of new active enzymes to compensate the loss of antioxidant activities which inhibited either by glycation or ROS due to STZ. In addition, the significant increase in gene expression of these genes might indicate that these enzymes were regulated at the transcriptional level. While some researches [39] demonstrated up regulation of gene expression of antioxidant enzymes in STZ diabetic rats, others indicated either no significant [28] or decrease [47] in activities and gene expression of theses enzymes in diabetic rats. Curcumin is known to augment antioxidant status especially through SOD [48,49]. Therefore, in the present study, by unknown mechanism, curcumin enhanced the gene expression of SOD which increased approximately 2 fold than control non diabetic and diabetic untreated rats. The significant elevation of SOD gene expression in diabetic rats treated with curcumin than diabetic untreated rats throughout the experiment indicated that, higher levels of superoxide radicals were produced which requires immediate dismutation [49]. The present findings come in accordance with previous results [50] which indicated that curcumin increased hepatic gene expression of SOD in chicken intoxicated with aflatoxin. Peroxisomal CAT and cytosolic GPX are involved in the conversion of H_2_O_2_ into water and molecular oxygen [51]. The significant increase of gene expression of CAT and GPX in diabetic rats treated with curcumin agrees with pervious results in chicken fed aflatoxin and treated with curcumin [50]. The insignificant difference of gene expression of CAT and GPX in untreated diabetic rats and diabetic rats treated with curcumin may be attributed to the synergistic effect of both enzymes for destruction of H_2_O_2_ produced by SOD or due to over expression of Bcl2 which is known as potent antioxidant. The significant elevation of GST gene expression in untreated diabetic rats as determined by real time PCR was to conjugate the reactive STZ metabolites with GSH as main step of detoxification process. The significant increase in gene expression of GST was more pronounced in diabetic rats treated with curcumin due to higher availability of GSH in this group. Although, administration of curcumin up-regulated the gene expression of antioxidant enzymes, the present study did not clarified the exact mechanism of action if it is due to direct or indirect effect of curcumin on the examined genes. Therefore, the present study recommends further investigation regarding this point.

IGF-1 has similar structures and functions like those of insulin particularly for peripheral uptake of glucose and fatty acids [17]. It is a single polypeptide with 70 amino acids and is widely expressed in mammal tissues [15-17]. The liver is the main source of circulating IGF-1 [52], thus the liver tissues were used for the expression study. IGF-1 induced its effect via binding to specific receptors on target cells. Insulin regulates liver IGF-1 expression either directly or indirectly by increasing the number of growth hormone hepatic receptors. Because of, IGF-1 induced glucose taken and improved the insulin sensitivity [53,54], increased its expression augmented this effect in diabetic rats treated with curcumin. Therefore, the increase of gene expression of IGF-1 by curcumin administration can be considered a new finding related to the present study, however, the actual mechanism of action could be subjected to further investigation. In the present study, liver tissue IGF-1 gene expression was not significantly changed in STZ diabetic rats compared to the control non diabetic which disagree with previous findings [18,55] indicating down regulation of IGF-1 in diabetic rats after acute or chronic administration of alloxan. This contradiction might be due to using different diabetogenic factor, experimental time and different RT-PCR protocols.

It well known that, the oxidative stress caused cell death via apoptosis. Bcl-2 is an integral mitochondrial membrane protein and acts as anti apoptotic and antioxidant factor [11]. Bcl-2 reduced the generation of ROS through binding to cytochrome *c* or prevents its entry to the cytosol [12]. There are a close relationship related the expression of Bcl2 and antioxidant enzymes, increase the expression of Bcl-2 exhibit elevated expression of antioxidant enzymes and higher levels of cellular GSH [13]. The significant increase in the expression of Bcl-2 in diabetic rats compared with control non diabetic rats was to counteract the oxidative stress arisen by diabetes. The significant up regulation of Bcl2 gene expression in diabetic rats treated with curcumin compare to diabetic untreated rats was to maintain the anti-apoptotic and antioxidant effect of Bcl2 in diabetic rats. This up regulation was concomitant with up regulation of IGF-1 which indicated that curcumin protected the body of diabetic rats via hypoglycemic and antioxidant effects. As discussed above, the anti hyperglycemic effect of curcumin was observed via up-regulation of IGF-1 gene expression in diabetic rats. Curcumin was antioxidant as reflected on up-regulation of gene expression and activities of antioxidant enzymes. Curcumin was antiperoxidative drugs as reflected on increased GSH contents with subsequent decrease in lipid peroxide biomarkers (TBARS). The anti-apototic effect of curcumin was also observed through up-regulation of Bcl2.

## Conclusion

The present study suggests that, curcumin can be used as natural hypoglycemic drugs in diabetic rats. The administration of curcumin reduced lipid peroxidation, elevated the glutathione capacity and increased the activities and gene expression of antioxidants enzymes. In addition, gene expression of IGF-1 and Bcl2 was also observed. Further studies are required to investigate whether the cause of activation of gene expression was due to a direct effect of curcumin in the studied enzymes or due to the overall effect of curcumin that leads to a cascade of reactions that resulting in the observed changes.

## Competing interests

The author declares that he has no competing of interests.

## Authors’ contribution

SME carried out experimental design, calculation of the experimental doses, induction of diabetes, oral administration of curcumin, blood and liver sampling, biochemical tests, RT-PCR analysis, real time RT-PCR analysis, statistical analysis, the manuscript draft and approved the final version of the manuscript.

## Pre-publication history

The pre-publication history for this paper can be accessed here:

http://www.biomedcentral.com/1472-6882/13/368/prepub
